# Application of Noninvasive Vagal Nerve Stimulation to Stress-Related Psychiatric Disorders

**DOI:** 10.3390/jpm10030119

**Published:** 2020-09-09

**Authors:** James Douglas Bremner, Nil Z. Gurel, Matthew T. Wittbrodt, Mobashir H. Shandhi, Mark H. Rapaport, Jonathon A. Nye, Bradley D. Pearce, Viola Vaccarino, Amit J. Shah, Jeanie Park, Marom Bikson, Omer T. Inan

**Affiliations:** 1 Department of Psychiatry and Behavioral Sciences, Emory University School of Medicine, Atlanta, GA 30322, USA; mwittbr@emory.edu (M.T.W.); mark.h.rapaport@emory.edu (M.H.R.); 2 Department of Radiology, Emory University School of Medicine, Atlanta, GA 30322, USA; jnye@emory.edu; 3 Atlanta VA Medical Center, Decatur, GA 30033, USA; ajshah3@emory.edu (A.J.S.); jeanie.park@va.gov (J.P.); 4 School of Electrical and Computer Engineering, Georgia Institute of Technology, Atlanta, GA 30332, USA; nil@gatech.edu (N.Z.G.); mobashir.shandhi@gatech.edu (M.H.S.); omer.inan@ece.gatech.edu (O.T.I.); 5 Department of Epidemiology, Rollins School of Public Health, Atlanta, GA 30322, USA; bpearce@emory.edu (B.D.P.); lvaccar@emory.edu (V.V.); 6 Department of Medicine, Cardiology, Emory University School of Medicine, Atlanta, GA 30322, USA; 7 Department of Medicine, Renal Medicine, Emory University School of Medicine, Atlanta, GA 30322, USA; 8 Department of Biomedical Engineering, City University of New York, New York, NY 10010, USA; bikson@ccny.cuny.edu; 9 Coulter Department of Biomedical Engineering, Georgia Institute of Technology, Atlanta, GA 30332, USA

**Keywords:** PTSD, stress disorders, posttraumatic, depressive disorders, vagus nerve, VNS, sympathetic, inflammation, interleukin-6, vagal nerve stimulation, interferon, stress

## Abstract

Background: Vagal Nerve Stimulation (VNS) has been shown to be efficacious for the treatment of depression, but to date, VNS devices have required surgical implantation, which has limited widespread implementation. Methods: New noninvasive VNS (nVNS) devices have been developed which allow external stimulation of the vagus nerve, and their effects on physiology in patients with stress-related psychiatric disorders can be measured with brain imaging, blood biomarkers, and wearable sensing devices. Advantages in terms of cost and convenience may lead to more widespread implementation in psychiatry, as well as facilitate research of the physiology of the vagus nerve in humans. nVNS has effects on autonomic tone, cardiovascular function, inflammatory responses, and central brain areas involved in modulation of emotion, all of which make it particularly applicable to patients with stress-related psychiatric disorders, including posttraumatic stress disorder (PTSD) and depression, since dysregulation of these circuits and systems underlies the symptomatology of these disorders. Results: This paper reviewed the physiology of the vagus nerve and its relevance to modulating the stress response in the context of application of nVNS to stress-related psychiatric disorders. Conclusions: nVNS has a favorable effect on stress physiology that is measurable using brain imaging, blood biomarkers of inflammation, and wearable sensing devices, and shows promise in the prevention and treatment of stress-related psychiatric disorders.

## 1. Introduction

Stress-related psychiatric disorders, including depression and posttraumatic stress disorder (PTSD), are important public health problems. Early life stress increases the risk of development of depression in adulthood [1,2], and stressful life events are associated with an increased risk for depressive episodes [3], while PTSD requires exposure to a traumatic stressor as part of the diagnosis [4]. At any given time, 10% of the United States population meets the criteria for major depression or other mood disorders based on Diagnostic and Statistical Manual (DSM) criteria [5], with an annual cost of lost productivity of USD 44 billion [6]. Similarly, PTSD affects 6% of the population at some time in their lives [7]. The cost of treating PTSD and comorbid depression in soldiers returning from the wars in Iraq and Afghanistan has been estimated to be USD 6.2 billion [8], and since PTSD affects a larger total number of civilians in the United States than military personnel, the costs for society as a whole are likely much higher [9]. The most common cause of PTSD in women is sexual abuse and assault in childhood, while, for men, it is physical assault [10]. On average, women have higher occurrence of PTSD compared to men in the civilian population [11,12]. PTSD is characterized by intrusive thoughts, nightmares, avoidance, emotional blunting, negative cognitions, hypervigilance, and hyperarousal [13]. Depression is associated with depressed mood, loss of appetite, decreased psychomotor activity, and, in extreme cases, suicidal ideation. Other symptoms, such as poor sleep and concentration, negative cognitions, loss of interest in things, and anhedonia, are common to both conditions. In fact, there is a degree of comorbidity between the two conditions [14,15,16,17,18,19]. Furthermore, patients with comorbid disorders have a worse clinical course, with, for instance, a higher risk of suicidal ideation [20,21].

The standard of care for both PTSD and depression includes psychotherapy and/or medication [22,23]. Psychotherapy treatments for PTSD, however, have dropout rates as high as 50%, which limit their applicability [24,25]. First-line medication treatments for stress-related psychiatric disorders involves the Selective Serotonin Reuptake Inhibitor (SSRI) antidepressants [26,27]. However, as highlighted by a report from the Institute of Medicine, there is not sufficient evidence to conclude that they are effective for PTSD [28]. In fact, only one-third of those suffering from PTSD are able to achieve full remission with the current standard of care [26]. Similar limitations exist for treatment of major depression. As illustrated by the STAR*D study, only one-third of patients with major depression remitted to first-line therapy with antidepressants and only about two-thirds of patients met remission criteria after multiple algorithms that included psychotherapy, switching classes, and multiple heroic augmentation trials [29]. Given limitations of current treatment options, new paradigms are clearly needed for the management of stress-related psychiatric disorders.

## 2. Physiology of the Vagus Nerve

**Figure 1:**
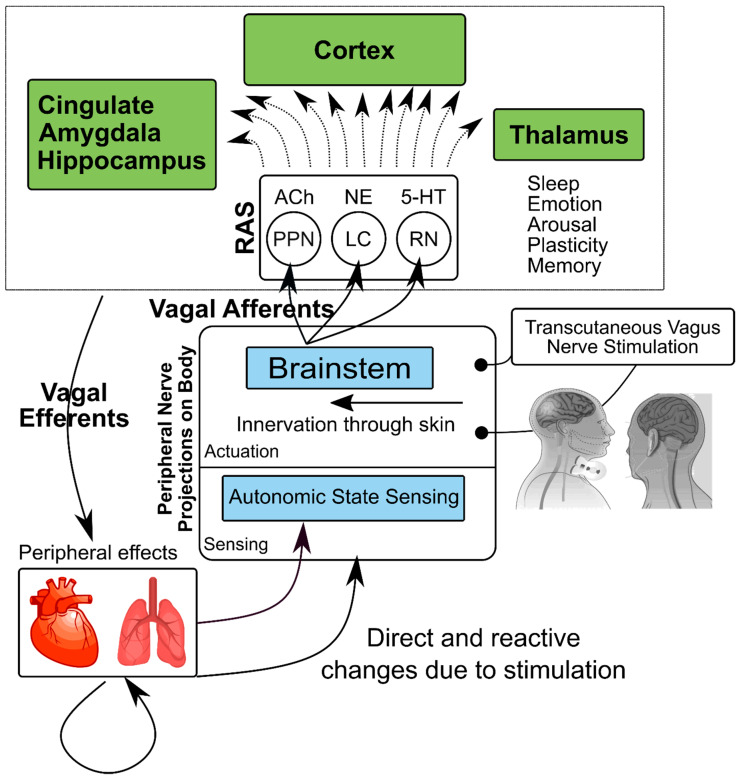
Model of effects of transcutaneous Vagal Nerve Stimulation (VNS) on physiological function. Stimulation of the vagus nerve in the neck as it passes through the carotid sheath (transcutaneous cervical VNS (tcVNS)) or in the ear (transcutaneous auricular VNS (taVNS)) activates the Nucleus Tractus Solitarius (NTS) in the brainstem, which has projections to other key brainstem nuclei containing cell bodies for neurotransmitters, including the locus coeruleus (LC), site of norepinephrine (NE), pedunculopontine nucleus (PPN) for acetylcholine (Ach), and raphe nucleus (RN) for serotonin (5-HT), and the reticular activating system (RAS). These regions, in turn, originate pathways to multiple brain areas involved in modulation of fear and emotion, as well as memory and neuroplasticity, including the anterior cingulate, hippocampus, amygdala, and cortex (including insula). Vagal efferents project to peripheral cardiovascular, autonomic, and inflammatory pathways. The vagus also projects information from the periphery back to the brain through afferents.

The vagus nerve represents a unique window between central functions of the brain and peripheral organ function that may be a promising target for treatment interventions for stress-related psychiatric disorders. The vagus has cell bodies in the brainstem and motor fibers that modulate peripheral organ function, as well as sensory fibers that relay information about peripheral organs to the brain (Figure 1). The efferent function of the vagus nerve primarily modulates parasympathetic nervous system function in the periphery and therefore acts as a counterbalance to the sympathetic nervous systems. Afferent vagal nerve fibers relay sensory activity of the visceral organs to the brain through the nucleus tractus solitarius (NTS) in the medulla oblongata and the locus coeruleus in the pons, with relays to areas of the brain involved in the modulation of emotion and the stress response, including the amygdala, insula, hippocampus, and anterior cingulate/prefrontal cortex [30].

## 3. Neurobiology of Stress-Related Psychiatric Disorders

The vagus nerve modulates circuits and systems that underlie the symptoms of stress-related psychiatric disorders. The neurobiology of stress-related psychiatric disorders includes alterations in brain regions involved in memory and the stress response [31,32,33,34]. The hypothalamic–pituitary–adrenal (HPA) axis plays an important role in stress, and dysregulation of this system is associated with PTSD and depression [35,36,37,38,39,40,41,42] and is potentially modifiable by the vagus nerve [43,44,45]. Norepinephrine and sympathetic function are also involved in stress-related psychiatric disorders, with elevations typically seen in both patients with PTSD and depression, in addition to dysregulation of the peripheral autonomic nervous system [46,47,48,49,50,51,52,53,54,55,56,57].

Abnormalities of the inflammatory function are also associated with PTSD and depression [58,59]. Interleukin 1B (IL1B), IL-6, Tumor Necrosis Factor (TNF), Interferon gamma (IFN-γ), and C-Reactive Protein (CRP) are elevated in PTSD [60], and several of these immune mediators are increased after acute stress [61,62]. Patients with cardiovascular disease and the diagnosis of PTSD had an enhanced IL6 response to stressful tasks (“mental stress”) compared patients without PTSD [63], with similar findings in individuals with early life stress, and vulnerability to depression [60,63,64]. Increased levels of proinflammatory cytokines and increased reactivity of the Nuclear Factor Kappa-light-chain-enhancer of activated B cells (NF-κB) system have been observed repeatedly in a subset of patients with depression [58,65]. Studies show that the stress-induced release of catecholamines acts through adrenergic receptors to activate NF-κB, and subsequently stimulates the release of cytokines, including IL-6 [66]. Interpersonal stress may have led to activation of inflammatory processes in response to imminent personal injury and therefore had survival value in evolution [58,67]. A recent meta-analysis showed that the statistically strongest findings in PTSD were for IL-6 and IFN-γ [68]. Elevations in IFN-γ and IL-6 are associated with decreases in tryptophan, the precursor of serotonin, a key neurotransmitter underlying the neurobiology of both depression and PTSD [69,70,71]. Increases in IL-6 also result in increases in kynurenine, which has been linked to suicide and depression [72], and quinolinic acid, which enhances glutamatergic transmission with associated decreases in brain derived neurotrophic factor (BDNF) in the hippocampus, which may be involved in symptoms of PTSD and depression and mechanisms of action of antidepressants [73,74,75,76]. These studies have shown the importance of intervention in the psychobiology of PTSD and depression, which potentially can be done with tcVNS.

Alterations in cell-mediated immunity in PTSD may be relevant to the mechanisms of action of tcVNS. Cell-mediated immunity utilizes T cells, including CD8+ cytotoxic cells that lyse cells harboring microbes and CD4+ cells that produce cytokines and activate phagocytes that engulf and kill microbes. These latter cells differentiate into Th1 and Th2 subsets, as well as Th17 subsets. T helper cell differentiation is partly controlled by cholinergic neurotransmission [77] and dysregulation of this system is associated with PTSD [78,79]. Glucocorticoids, including cortisol, inhibit immune function and lower concentrations of cortisol in patients with PTSD [41,80], and could result in enhancement of Th1 cell function [78,79] with an associated increase in IFN-γ. Studies have shown enhanced cell-mediated immunity in PTSD patients [81] and delayed-type hypersensitivity (DTH) reactions that are consistent with an enhancement of Th1 response and thus increased IFN-γ [82]. Other studies have linked DTH responses to elevated IFN-γ [83] and have shown increased IFN-γ in PTSD [68,84,85]. Vagus nerve stimulation activates T cells that produce acetylcholine, and by binding to the alpa-7 subunit of the cholinergic receptor, they inhibit NF-κB [86], and, based on our studies, IFN-γ [87]. VNS also inhibits High Mobility Group Box 1 protein (HMGB1), a proinflammatory master mediator, which is increased in PTSD and is modifiable by VNS [88,89]. These studies have shown several targets for modulation of immune function by vagal nerve stimulation in the treatment of stress-related psychiatric disorders.

Altered neuropeptidal function is another target for treatment intervention for stress-related psychiatric disorders [33]. Pituitary adenylate cyclase activating polypeptide (PACAP) is a neuropeptide that regulates and integrates adaptive responses to stress [90]. A growing body of literature has pointed to dysregulation of PACAP along with its selective PAC1 receptor in PTSD [90,91]. Elevated PACAP levels were associated with increased PTSD symptoms in females with PTSD [91]. In other studies, PAC1 receptor levels correlated with increased startle response [92,93], a marker of PTSD [94,95,96]. PACAP plays an important role in physiological stress responses, including those mediated by the sympathetic and parasympathetic nervous system [97]. PACAP distribution in brain areas involved in stress and emotion, including the hypothalamus, bed nucleus of the stria terminalis, and amygdala, suggests PACAP’s involvement in limbic, autonomic, and neuroendocrine functions [98,99]. These systems are also regulated by the vagus nerve, an effect possibly mediated by PACAP.

## 4. Neuromodulation for Stress-Related Psychiatric Disorders

Neuromodulation represents a promising new paradigm for the treatment of stress-related psychiatric disorders [100]. Neuromodulation involves the use of electricity, magnetism, vibration, or ultrasound actuation to modulate neural function [101]. Forms of neuromodulation that have been applied to psychiatry include electroconvulsive therapy (ECT), transcranial magnetic stimulation (TMS), transcranial Direct Current Stimulation [102], and Vagal Nerve Stimulation (VNS) [103,104,105,106,107]. Electrical stimulation treatments show promise for the treatment of stress-related psychiatric disorders since they may act through effects on the underlying neurobiology of these disorders [103,104,106,108,109].

Electroconvulsive therapy (ECT) is one of the most effective treatments for patients with treatment-refractory major depression [110]. ECT involves the application of electrical currents to the skull while patients are under anesthesia with the goal of inducing a seizure with associated multiple firing of neurons felt to lead the therapeutic effect seen after multiple treatments [111]. ECT has an 80% response rate, which is a better response rate than for medications [110]. Some studies have suggested that ECT may be more effective in elderly depressed patients [112]. Predictors of good response include low Vitamin B-12 and folate levels and elevated homocysteine (all of which have been linked to depression) [113]. ECT may induce mechanisms which, as reviewed below, have been posited for VNS, and which are common to other successful antidepressant treatments, including modulation of excitatory amino acid transmission and promotion of neurogenesis in the hippocampus [75] and modulation of brain function in the medial prefrontal cortex [114]. ECT results in profound hemodynamic changes including bradycardia, followed by tachycardia and hypertension, as well as increased complication rates, can occur in patients with cardiovascular disease [111]. In spite of relative safety, many patients are hesitant to use ECT due to inconvenience and fear of side effects.

VNS is an electrical stimulation treatment that, in its implantable form, has been shown to be efficacious in the treatment of epilepsy [115,116,117,118,119,120] and treatment-refractory major depression [121,122,123,124,125,126,127,128,129,130,131,132], leading to approval by the Food and Drug Administration (FDA) for the treatment of these conditions [133]. VNS, which is currently approved for depression, involves surgical implantation with direct electrical stimulation of the vagus nerve [124,134,135].

VNS has a number of effects on brain circuits and systems that are likely beneficial for stress-related psychiatric disorders. In experimental models of PTSD, vagus nerve stimulation enhanced extinction of conditioned fear and reduced PTSD-like symptoms [136,137]. Thus, studies suggest that VNS is potentially useful for stress-related psychiatric disorders.

VNS has effects on autonomic nervous system function that are likely beneficial for stress-related psychiatric disorders [138,139]. VNS improves autonomic dysfunction, reducing sympathetic and enhancing parasympathetic tone [140,141,142,143]

VNS has other effects, including the modulation of fear circuits [137,144,145], induction of neural plasticity [140], enhancement of memory and cognition [130,146,147,148,149,150,151,152,153,154,155,156], and enhancement of central neurotransmitter function including norepinephrine [157,158]. VNS also reduces inflammatory function [159,160,161,162,163,164,165]. These findings suggest that VNS may be useful for stress-related psychiatric disorders characterized by central neurotransmitter and peripheral autonomic dysfunction, enhanced inflammation, and impairments in learning and memory [46,47,49,50,51,52,53,54,166].

## 5. Vagus Nerve and Neuroplasticity

In addition to efferent fibers, the vagus has afferent fibers that modulate central brain function. Effects of the vagus on neural function include both inhibition of cortical spreading depression [167], as well as effects on brain amino acids, neurotransmitters, and metabolites [168,169,170]. The vagus also has effects on neural plasticity, as evidenced by enhancement of recovery following cerebral hemorrhage with vagal nerve stimulation [171]. Pairing of vagal nerve stimulation with an auditory tone was beneficial in an animal model of tinnitus, a disorder involving ringing in the ears, an effect mediated through enhancement of neural plasticity in relevant areas of the brain [172,173,174,175]. The vagus enhances neural plasticity after stroke, with beneficial effects both for the recovery of cognitive function [176], as well as motor movement when paired with training in successful motor movements [177,178,179,180]. The enhancement of new learning and memory following stimulation of the vagus [149,181] probably occurs through enhancement of long term potentiation (LTP) in the hippocampus [182]. The vagus also modulates fear circuits in the brain in a way that promotes adaptive stress responses [46,136,137,144,183]. In addition to its effects on the brain, the vagus promotes recovery following cardiovascular events in animal models [139,184,185,186].

## 6. Neural Circuits in Stress-Related Psychiatric Disorders and Vagal Nerve Stimulation

Understanding neural circuits in stress-related psychiatric disorders is important for designing new treatments such as VNS that can target these underlying neurobiological disturbances [187,188]. A network of brain areas, including the hippocampus, amygdala, insula, and medial prefrontal cortex (including anterior cingulate), have been implicated by us and others in the pathophysiology of stress-related psychiatric disorders [34]. The hippocampus, which plays a critical role in declarative (or explicit) memory, is very sensitive to stress [75,189,190,191,192]. These effects are reversible with treatment with antidepressants or behavioral interventions like running [73,75,76]. Studies in patients with both PTSD and depression have shown alterations in memory function and reduction of hippocampal volume [193,194,195,196,197]. The amygdala is involved in the processing of emotional stimuli and emotional memory, and plays a critical role in the acquisition of fear responses [198]. The medial prefrontal cortex/anterior cingulate has been implicated in the appraisal and regulation of emotions, and inhibition of the amygdala function represents the mechanism of extinction [199]. Brain imaging studies have implicated the medial prefrontal cortex/anterior cingulate in PTSD [200,201,202,203,204,205,206,207,208,209,210,211,212,213,214,215] and depression [216,217,218]. An increase in function in PTSD is observed in insula [205,209,219] and increased amygdala function is associated with both PTSD [204,205,213,215,220,221,222,223,224,225,226,227,228,229,230,231,232,233,234,235,236,237] and depression [238,239,240,241]. Treatment is associated with changes in these brain regions for both PTSD [34,242,243,244,245,246,247] and depression [240,241,248,249,250,251,252]. As reviewed below, similar studies have been performed looking at neural circuits and systems response in depression [253,254,255,256,257,258,259], and our group has initiated studies using High-Resolution Positron Emission Tomography (HRPET) to assess neural correlates of treatment response in PTSD [260].

## 7. Noninvasive Vagal Nerve Stimulation: Safety and Reliability

Recently, devices have been developed for noninvasive stimulation of the vagus nerve [261]. Noninvasive Vagal Nerve Stimulation (nVNS) devices include transcutaneous auricular VNS (taVNS), which target the auricular branch of the vagus in the ear (with best results at the cymba conchae and tragus) [262], and transcutaneous cervical VNS (tcVNS), which act on the cervical branch as it passes through the carotid sheath in the neck [100]. These devices have been shown to be safe and effective and to reliably and predictably stimulate the vagus nerve in human subjects [263,264]. Vagal somatosensory evoked potentials associated with vagal afferent activation have been reported for both implanted VNS and noninvasive VNS devices used through the neck or ear [265]. Studies using evoked potentials have shown that nVNS reliably stimulates the vagus nerve in humans and anesthetized dogs [141,266,267,268]. Studies in humans using electroencephalography (EEG) measurements at scalp sites A1-Cz showed that tcVNS and taVNS both result in a predictable and reproducible P1 N1 P2 N2 pattern with biphasic peaks at 3 ms (P1, N1), followed by a large stimulation artifact and large biphasic peaks (P2, N2) at 10 ms that matches the pattern seen with implanted VNS [265,266,268]. Functional brain imaging studies of healthy human subjects with nVNS, including both taVNS [262,269,270] and tcVNS [271], showed the characteristic pattern of neural response of brain areas known to be connected to the Nucleus Tractus Solitarius (NTS), the primary relay point for vagal nerve fibers to the brain. These studies are all consistent with nVNS resulting in stimulation of the vagus nerve with resultant central effects in the brain. Other studies of neurobiology are also consistent with the role of nVNS in stimulating the vagus nerve.

NVNS has effects on a range of stress-related biological parameters. Studies using taVNS showed improved vagal activity [272,273], increased salivary alpha amylase, and decreased salivary cortisol [274]. Psychophysiology-focused studies have produced mixed outcomes. Whereas some studies noted no effect of taVNS on physiological markers of autonomic activity, such as pupil size, startle blink electromyography, and skin conductance responses [274,275,276], other studies noted improvements in psychophysiological indices of vagal activity with taVNS [272,273]. Our group recently studied tcVNS in physically healthy human subjects with a three-day stress paradigm, and found favorable results, indicating decreased physiological reactivity during stress and at rest for a wide range of biomarkers that could be obtained with wearable sensing devices [277,278]. We also explored computational methods to determine stimulation presence based on these continuous assessments of autonomic activity [279]. Anti-inflammatory effects of tcVNS based on serum cytokines have been noted in different healthy human studies and patients with Primary Sjögren’s Syndrome [280,281,282].

## 8. Noninvasive Vagal Nerve Stimulation: Application to Stress-Related Psychiatric Disorders

The requirement for direct VNS to be surgically implanted has limited widespread implementation in stress-related psychiatric disorders to date due to cost and inconvenience [125,131]. These forms of VNS are also limited by the fact that true sham-controlled trials cannot be performed due to ethical reasons, which has led to questions about the true efficacy of these devices [261]. Since devices are only implanted in patients who have not responded to multiple antidepressants, the patient populations are also not necessarily representative of those typically seen in clinical psychiatry practices, which may explain why VNS, although yielding statistically significant improvements, did not lead to complete remission in all patients [283]. Additionally, treatments have not been reimbursed by Medicare or other insurance companies, which has further limited implementation [284]. Studies have shown the utility of both tcVNS and taVNS for various psychiatric disorders, including schizophrenia [285] and obsessive-compulsive disorder [286], as well as major depression [287]. Human studies also suggest that noninvasive VNS improves hyperarousal in PTSD patients with mild traumatic brain injury [288] and reduces symptoms in treatment-resistant anxiety disorders [289].

As proven by their cost and convenience, noninvasive VNS technologies have widespread applicability to patients with stress-related psychiatric disorders.

**Figure 2:**
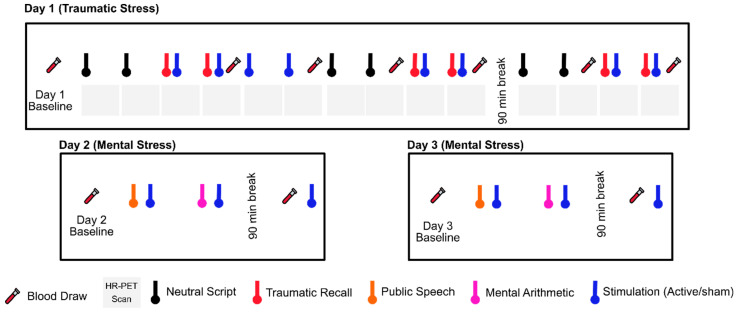
Study protocol undergoing since 2017. Physiological sensing data is collected continuously throughout three study days. The protocol timeline depicts neutral and trauma scripts, HR-PET scans (first day), mental stress tasks of public speech and mental arithmetic (second and third day), stimulation with active tcVNS or sham, and blood draws (all days).

Neck-based tcVNS was recently approved by the FDA for the treatment of intractable cluster headache [264,290,291,292]. We have implemeneted this device in healthy human subjects with a history of exposure to traumatic stressful events since 2017, and have found it to be safe and feasible [277,278,279]. In our studies, we compared active tcVNS to a sham control in a randomized trial (Figure 2). Both were handheld devices that were applied to the left neck for stimulation with identical placement and operation (GammaCore, ElectroCore, Basking Ridge, New Jersey). nVNS or sham were applied using collar electrodes on the left side of the neck in order to permit placement while subjects were in the research-dedicated brain scanner, which had a small aperture. The treatment area on the neck was located by finding the carotid artery pulsation. An electrically conductive gel was applied on the stimulation surfaces and device is placed on the located treatment area. Active tcVNS devices produced a 5-kHz sine wave burst lasting for 1 millisecond (five sine waves, each lasting 200 microseconds), repeated one in every 40 milliseconds (25 Hz), generating 30-V peak voltage and 60-mA peak output current. The final stimulation intensity depended on the subject’s verbal feedback: The researcher was instructed to increase the intensity gradually until the subject voiced discomfort, at which point the intensity was reduced slightly below that threshold. Sham devices produced a nearly direct voltage signal, whose polarity was slowly varied (0.2-Hz biphasic voltage), in contrast to the higher-frequency, alternating current used for the active nVNS (25 Hz with 5-kHz bursts). The sham device delivered a biphasic signal generating a 14-V peak voltage and 60-mA peak output current, consisting of pulses repeating every 5 s (0.2 Hz). High-frequency voltage signals (such as the active stimulus) pass through the skin with minimal power dissipation due to the low skin-electrode impedance at kHz frequencies. In contrast, lower-frequency signals (such as the sham stimulus) are mainly attenuated at the skin-electrode interface due to the high impedance [293]. Accordingly, the active tcVNS can deliver substantial energy to the vagus nerve to facilitate stimulation, while the voltage levels appearing at the vagus would be expected to be orders of magnitude lower for the sham device and thus vagal stimulation is unlikely. Nevertheless, since the sham device does deliver relatively high voltage and current levels directly to the skin, it activates skin nociceptors, causing a similar feeling to a pinch. This sensation is necessary for blinding of the participants and is thought as a critical detail by the investigators for the valuation of the potential treatment in psychiatric populations. Both active and sham interventions lasted for two minutes. The subject, research staff, and investigators were all blind to the device category, and the key was kept in a locked office by an individual not involved in the research in two locations. The specific details are summarized as follows: The manufacturer sent the active and sham devices to an individual who was not involved in research, and the individual randomized patients to the devices prior to patient recruitment. In addition, every subject was given a different, dedicated device, hence the number of patients was equal to the number of devices. Every week when a new patient arrived, the individual not involved in research delivered a different device to the research staff for use for that subject.

In our study with physically healthy traumatized subjects and patients with PTSD, we constructed a multisignal dataset that include physiological signals related to cardiovascular and peripheral activity. The signals included electrocardiography (ECG), respiration (RSP), seismocardiography (SCG), photoplethysmography (PPG), electrodermal activity (EDA), and blood pressure (BP). Upon beat-by-beat signal processing, we extracted parameters related to autonomic tone with a beat-by-beat resolution. These parameters included both standard and nonstandard indices of psychophysiological reactivity, such as heart rate (HR), pre-ejection period of the heart (PEP), amplitude of the peripheral photoplethysmogram (PPG), pulse arrival time (PAT), properties of respiration signal (respiration rate, RR, width, RW, prominence, RP), frequency- and time- domain heart rate variability indices including low- and high-frequency heart rate variability (LF HRV, HF HRV), Poincare-based nonlinear heart rate variability (SD1, SD2), acceleration and deceleration capacity (AC, DC), and skin conductance level and response (SCL, SCR). In our healthy cohort, PEP, PPG amplitude, skin conductance, and respiratory indices resulted in marked differences between active and sham groups, indicating a blunted sympathetic response with tcVNS [277,294]. We later used this blunted physiological reactivity pattern to devise a machine learning based method that could indicate stimulation presence [278,279]. Brain imaging using High-Resolution Positron Emission Tomography (HR-PET) in traumatized participants without PTSD exposed to personalized traumatic scripts showed that tcVNS compared to sham stimulation blocked activations in the medial prefrontal cortex, parahippocampal gyrus, and insula, brain areas that play key roles in emotion and response to stress [295].

We also studied the effects of tcVNS on inflammatory markers in traumatized individuals with and without PTSD. We found that tcVNS paired with personalized traumatic scripts blocked stress-induced increases in proinflammatory biomarkers IL-6 and IFN-γ, and showed a pattern of decreased anger responses to scripts [87]. Increases in IL-6 and IFN-γ likely occur multiple times a day with minor stressors and triggers in PTSD patients, so tcVNS could result in a decrease in symptoms driven by inflammation and lead to improvements in clinical course. The reduction in subjective anger, in addition to improved mental health, also likely have beneficial health effects, for instance, in patients with comorbid PTSD and coronary artery disease (CAD), where we found not only an increase in mental stress-induced IL-6 in those with comorbid PTSD [63], but also that anger, PTSD, and other symptoms of psychological distress were associated with long-term adverse cardiovascular outcomes [296] and an increase in mental stress-induced myocardial ischemia [297,298].

Studies are ongoing with patients with PTSD, paired with assessment of the brain with High Resolution Positron Emission Tomography (HR-PET), and assessment of inflammatory and other blood biomarkers [278]. Due to low cost, increased convenience, limited side effects, feasibility for use at home or in the field for military medicine applications, and the ability to assess efficacy with true sham control comparison, tcVNS and taVNS show great promise in our opinion for the treatment of patients with stress-related psychiatric disorders and enhancement of human performance [261].

## 9. Conclusions

Current treatments for PTSD, major depression, and other stress-related psychiatric disorders, including medications and psychotherapy, have limitations and are not efficacious for all patients. Neuromodulation is an important alternative treatment, and noninvasive forms of VNS have the advantages of cost and noninvasiveness and can potentially be widely implemented for these patients. Both tcVNS and taVNS show promise for intervening at the level of the underlying neurobiology of these disorders.

PTSD is triggered by experiencing or witnessing exposure to traumatic events and leads to uncontrollable thoughts about the events. Our results from traumatized subjects without PTSD demonstrate decreased sympathetic and increased parasympathetic tone during tcVNS following acute traumatic stress, suggesting possible translation of this treatment to patients with PTSD, in the clinic or at home, as an acute treatment for these recurrent memories [277,278,279,299]. tcVNS has potential promise for enhancing recovery from acute traumatic stress by means of modulation of autonomic response in PTSD populations. As patients with PTSD show exaggerated responsivity to reminders of traumatic memories, the physiological changes induced by tcVNS observed in traumatized individuals without PTSD may be similarly observed in PTSD populations. Moreover, recent studies have shown that invasive VNS enhances the extinction of conditioned fear in rats [137]. Additionally, taVNS was shown to lead to improvement in vagal tone in patients with PTSD [288] and to inhibit long-term fear responses during extinction training in healthy human subjects [300]. Implanted VNS has already been approved by the FDA as a treatment for treatment resistant depression and epilepsy, but its cost and the intrusive nature of the surgery have limited its use. Noninvasive VNS technologies would be a significant addition to both facilitate further research into the circuitry of PTSD and treatment resistant depression, and would provide a new and highly acceptable treatment option for patients suffering from both severe and recurrent depression and PTSD [134,261].
